# Physical Fitness in Upper Austrian Children Living in Urban and Rural Areas: A Cross-Sectional Analysis with More Than 18,000 Children

**DOI:** 10.3390/ijerph17031045

**Published:** 2020-02-07

**Authors:** Clemens Drenowatz, Franz Hinterkörner, Klaus Greier

**Affiliations:** 1 Division of Physical Education, University of Education Upper Austria, 4020 Linz, Austria; 2 Olympic Training Center Upper Austria, 4020 Linz, Austria; 3 Division of Physical Education, Private Educational College (KPH-ES), 6422 Stams, Austria; 4 Department of Sport Science, Leopold-Franzens-University Innsbruck, 6020 Innsbruck, Austria

**Keywords:** living environment, cardiorespiratory endurance, muscular strength, motor skills, body weight, youth

## Abstract

Physical fitness is an important component in the development and health of children and adolescents. Given the equivocal results of previous research regarding the influence of the living environment on physical fitness, this study examined differences in physical fitness in urban and rural elementary school children in Upper Austria. A total of 18,168 (51% male) children between 6 and 11 years of age participated in anthropometric assessments and completed eight fitness tests that assessed cardiorespiratory endurance, muscular power, speed, agility, flexibility, and ball handling skills during a single test session in the school’s gymnasium. Urban living environment was associated with higher body weight (*p* = 0.01) and lower physical fitness (*p* < 0.01), except for flexibility, which was better in urban children (*p* < 0.01) and upper body strength (no difference), even after accounting for differences in body weight. Furthermore, it was shown that urban–rural differences in physical fitness are more pronounced in normal weight children and that these differences increase with age. These results highlight the potential of the living environment for the promotion of an active lifestyle that enhances physical fitness. The availability of safe spaces that facilitate unstructured and structured physical activity, as well as active transportation provide viable options for the promotion of physical fitness in children.

## 1. Introduction

Over the last several decades, there has been a trend towards an increased concentration of the population in cities, which is referred to as urbanization [1,2]. This change in residential pattern reflects an economic transition towards a service-based economy, which also influences biological development and behavioral choices of human beings. Urban living environment, for example, has been associated with accelerated physical growth and sexual maturation [3,4]. Furthermore, research has indicated differences in behavioral choices such as eating habits and physical activity (PA) between urban and rural residents [5,6,7,8,9]. Specifically, urban adolescents have been shown to consume more fast food and alcohol [9], which potentially contributes to a higher energy intake. Regarding PA, urbanization has been associated with lack of space for play, safety concerns, and passive transportation that contribute to a more sedentary lifestyle in children, despite a potentially better access to sports facilities in urban areas [5,6,10,11,12]. An increase in inactive habits, such as reading, playing computer games, and watching TV is also associated with a reduction in time spent outdoors [10,13], which has been shown to be an important correlate of habitual PA [14,15] and further emphasizes the role of the environment in facilitating an active lifestyle. Low PA, most likely, also contributed to the observed decline of physical fitness in youth [16,17,18]. As physical fitness is defined as a person’s ability to carry out daily activities without undue fatigue and adequate energy reserves to pursue and enjoy recreational pursuits [19], physical fitness can have profound effects on habitual PA. Furthermore, there is an independent association of physical fitness with chronic disease risk [20,21,22], cognitive development, and academic performance [20,23,24]. Accordingly, physical fitness at young ages is of critical importance for various health outcomes and overall quality of life [25,26].

Various studies have examined urban–rural differences in PA and physical fitness, including cardiorespiratory endurance, muscular strength, muscular endurance, and flexibility in children and adolescents. Results, however, have been inconsistent. Some studies have shown higher PA and physical fitness levels in urban youth [27,28,29], while others have shown opposite results [2,30,31]. There is also research indicating limited differences in physical fitness by living environment [6,32,33]. These inconsistencies could be attributed to differences in the definition and criteria used to differentiate between urban and rural living environments, as well as the methodology used to assess PA and physical fitness. Furthermore, environmental differences such as climate or features of the built environment in urban and rural settings could have contributed to the variability in findings across countries. In addition, economic, cultural, and social factors that potentially affect availability and access to PA facilities that promote physical fitness along with the perception of the environment and differences in policies (e.g., mandatory physical education (PE) in schools) potentially affect the association between living environment and physical fitness.

In Austria, children engage in mandatory PE for two to three lessons per week. Opportunities for school sports, however, are limited and school generally lasts only until early afternoon. Accordingly, free play during leisure time and participation in club sports are key contributors to PA in children and adolescents. On the one hand, given that club sports are predominantly organized by volunteers, the availability of club sports for children are limited in rural communities. More open spaces for free play in rural communities, on the other hand, provide more opportunities for unstructured PA. Given the limited information on the association of living environment with physical fitness in Austrian youth, this study examines urban–rural differences in various components of physical fitness in a large, relatively homogenous, sample of Upper Austrian elementary school children. It was hypothesized that rural elementary school children display higher physical fitness as compared with their urban peers because unstructured PA has a greater influence on fitness than structured PA at this age.

## 2. Materials and Methods

In order to promote PA and motor competence in elementary school children the Upper Austrian government initiated the project “Wie fit bist du?” (i.e., How fit are you?), in 2016, which consisted of annual fitness assessments at participating elementary schools. All schools in Upper Austria received information about the project and could, subsequently, volunteer for participation. Until July 2019, a total of 28,481 fitness tests were completed in more than 200 participating schools. As several schools participated in consecutive years, there were several children with multiple measurements throughout the three-year observation period. In order to avoid duplicate cases, in this study, the analyses, therefore, used only data from the first fitness assessment of participating children, which resulted in a final sample of 18,168 (51% male) children between six and 11 years of age. Study procedures were approved by the Upper Austrian School Board and written informed consent was obtained from participating schools and parents. Children provided oral assent at the time of measurement.

All assessments were performed by trained technicians in the participating school’s gymnasium. Anthropometric measurements were taken according to standard procedures with children in gym clothes and barefoot. Body height was measured with a portable stadiometer (SECA 2013, Seca, Hamburg, Germany) to the nearest 0.5 cm and body weight was measured with an electronic scale (Seca 878 dr, Seca, Hamburg, Germany) to the nearest 0.1 kg. Subsequently, BMI was calculated (kg/m^2^) and converted to BMI percentiles (BMIPCT) using German reference values [34]. Children with a BMIPCT above the 90th percentile were classified as overweight/obese.

### 2.1. Fitness Assessment

Following a standardized 5 min warm up, children completed 8 fitness tests within 90 to 120 min that assessed ball handling skill, flexibility, agility, speed, muscular power, and cardiorespiratory endurance. Specifically, ball handling skill was assessed via the number of catches of a ball thrown against the wall from 1.5 m distance within 30 s. Performance at the stand-and-reach test was used as an indicator for flexibility. Agility was assessed by the time needed to complete a standardized obstacle course (Hürden-Bumerang Lauf) [35] that included a forward roll and jumping over as well as moving under hurdles along with directional changes. Speed was assessed with a 10 m sprint (timed via photocell measurement; TDS Linz, Austria) as well as the as the number of contacts during a 6 second tapping task on a force plate (TDS, Linz, Austria). Vertical jump performance (assessed via force plate) and distance of a medicine ball toss were used as indicators for muscular power. Distance covered during a 6 min run was used as indicator for cardiorespiratory endurance. All tests were verbally explained and shown to the participants prior to the respective assessments. Participants performed each test twice, except for the vertical jump (3 trials) and the 6 min run (1 trial) and the best performance scores were used for data analysis. The fitness assessments were completed in random order, except for the 6 min run, which was completed at the end of the testing session in order to minimize fatigue throughout the testing session.

### 2.2. Living Environment

Urban or rural status was determined based on the urban-rural typology of the Austrian Statistics Agency [36]. Out of 440 Upper Austrian communities, 34 are classified as urban centers that cluster in 4 areas. Roughly 38% of the Upper Austrian population (36% aged under 15 years) live in urban centers [36]. As children generally attend elementary school in their local community, children attending elementary schools in urban centers were considered living in an urban environment while children attending elementary schools in rural communities were considered living in a rural environment. In addition, participants were stratified into 3 age groups (<8 years of age, 8 to 9 years of age, >9 years) to further explore the association of living area with physical fitness.

### 2.3. Statistical Analyses

Descriptive statistics were calculated, and data was checked for normal distribution. Differences between children living in urban and rural areas were initially checked via MANOVA. Given the influence of age, sex, and BMIPCT on physical fitness, these variables were included as covariates in a subsequent MANCOVA. Furthermore, a 2 (urban/rural) × 3 (age group) MANCOVA, adjusting for sex (and subsequently for BMICPT), was performed to check for age-by-living area interaction effects. Similar analyses were additionally performed separately for boys and girls, as well as for non-overweight and overweight/obese children. All statistical analyses were performed in SPSS 24.0 with a significance level of *p* < 0.05 and Bonferroni adjustment for multiple comparisons.

## 3. Results

Of the 18,168 participants, 22.7% attended schools in urban communities. There was no sex difference between the urban and rural study population (50.5% vs. 51.6% male, respectively). Urban participants, however, were slightly younger than rural participants and this difference was significant (*p* < 0.01) (Table 1). The mean BMIPCT of urban children was significantly higher as compared with their rural peers (*p* = 0.01) and more urban children were classified as overweight/obese (15.8% vs. 14.3%, *p* = 0.02). Rural children performed better than urban children at vertical jump, tapping, 10 m sprint, agility run, 6 min run, and throw and catch task (*p* < 0.01). Flexibility, indicated by the distance reached beyond the toes during the stand-and-reach test, was higher in urban children as compared with their rural peers (*p* < 0.01). No urban–rural difference was observed at the medicine ball push (*p* = 0.16). These results remained essentially unchanged after adjusting for age, sex, and BMIPPCT.

**Figure 1:**
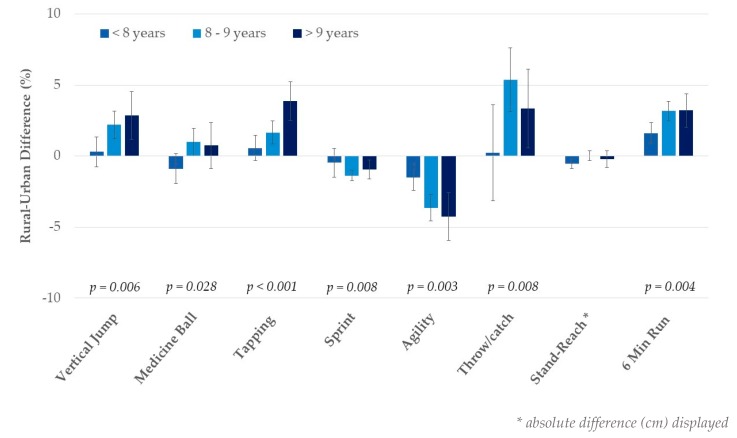
Rural–urban differences by age group. Values are mean differences, adjusted for sex with 95% CI, and *p*-values reflect significance for age-by-living area interaction.

Performance on the physical fitness tests improved with increasing age in the entire sample (*p* for trend across age groups <0.01). Age-by-living area MANCOVA, however, also showed significant interaction effects across all fitness test items (*p* < 0.03, adjusted for sex), except for flexibility. As shown in Figure 1, urban–rural differences in physical fitness were limited at younger ages and became more pronounced with increasing age. For flexibility, however, a significant urban–rural difference was observed in children under the age of eight but not in older participants. These results remained essentially unchanged after additionally adjusting for BMIPCT.

Although BMIPCT was significantly higher in boys as compared with girls (52.9 ± 29.4 vs. 50.7 ± 30.1, *p* < 0.01), boys displayed better physical fitness than girls (*p* < 0.01), except for flexibility, which was higher in girls (*p* < 0.01). Urban girls were heavier than their rural peers (BMIPCT 52.0 ± 30.4 vs. 50.3 ± 30.0, *p* = 0.03) while there was no urban–rural difference in BMIPCT among boys (53.7 ± 29.4 vs. 52.7 ± 29.3, *p* = 0.18). Rural girls displayed significantly better physical fitness (*p* < 0.01), except for flexibility, which was better in urban girls (*p* = 0.02), and the medicine ball push, even after accounting for differences in BMIPCT. In boys, urban–rural differences were only observed for speed, agility, and endurance-related tasks (*p* < 0.01). Ball handling skills, upper body strength, and flexibility did not differ between urban and rural boys (Table 2).

Across the entire sample, non-overweight children performed better at vertical jump (20.4 ± 3.7 vs. 16.9 ± 3.3 cm), tapping (45.2 ± 7.5 vs. 43.7 ± 7.3 contacts in 6 s), 10 m sprint (2.3 ± 0.2 vs. 2.4 ± 0.2 s), agility test (19.5 ± 3.2 vs. 22.8 ± 4.6 s), stand-and-reach (1.8 ± 6.6 vs. 1.0 ± 7.0 cm beyond toes), and 6 min run (1002 ± 125 vs. 865 ± 126 m) as compared with overweight/obese children (*p* < 0.01). Overweight/obese children performed better at the medicine ball push (381 ± 77 vs. 345 ± 72 cm, *p* < 0.01) as compared with their non-overweight peers, whereas there was no significant difference at the throw and catch task (15.9 ± 7.5 vs. 15.3 ± 7.7). When non-overweight and overweight/obese participants were analyzed separately, urban–rural differences were significant in most components of physical fitness in non-overweight participants, while in overweight/obese participants differences were only significant for the 20 m sprint, agility test, and the 6 min run. These results remained after adjusting for age and sex (Table 3).

**Figure 2:**
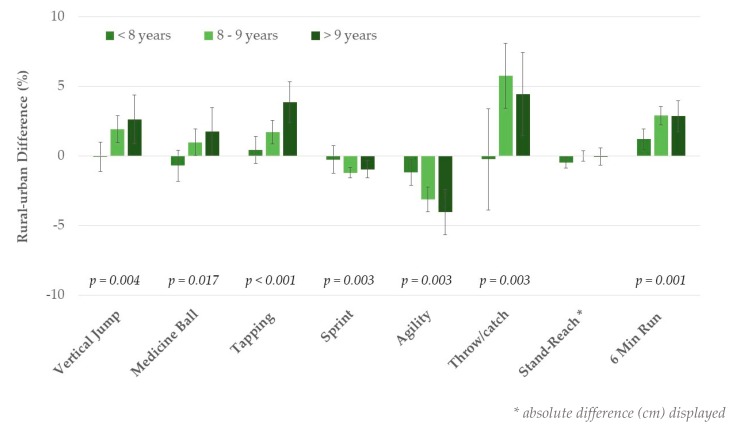
Rural–urban differences by age group in non-overweight children. Values are mean differences, adjusted for sex with 95% CI, and *p*-values reflect significance for age-by-living area interaction.

**Figure 3:**
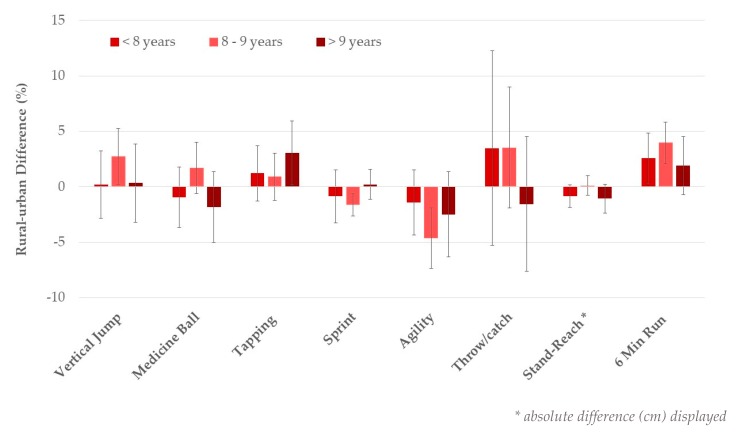
Rural–urban differences by age group in overweight/obese children. Values are mean differences, adjusted for sex with 95% CI.

In addition, there were significant age-by-living area interaction effects across all physical fitness tests in non-overweight participants (*p* ≤ 0.02, adjusted for sex), except for the stand-and-reach test. As shown for the total sample, urban–rural differences became more pronounced with increasing age, except for flexibility (Figure 2). No significant age-by-living area interaction effects were observed in overweight/obese children (Figure 3).

## 4. Discussion

This study extends previous research on the association between living environment and health by examining urban–rural differences in various components of physical fitness in Upper Austrian elementary school children. As shown in previous studies, living environment appears to influence body composition and physical fitness [28,29,30,31]. The results of this study showed a lower BMIPCT and higher physical fitness in rural elementary school children as compared with their urban peers, except for flexibility, which was better in urban children, and medicine ball push in which no urban–rural differences were observed. Although physical fitness is inversely associated with body weight [37,38], urban–rural differences in physical fitness remained after accounting for differences in body weight. The higher flexibility in urban children could be attributed to a greater participation in club sports such as gymnastics or dancing, which are limited in rural settings due to the greater popularity of ball sports (e.g., soccer and tennis). The results of this study further show that urban–rural differences in physical fitness become more pronounced with increasing age, particularly, in normal weight children. In addition, boys displayed higher physical fitness than girls already at young ages and urban–rural differences were more pronounced in girls.

Although results on the association between living environment and physical fitness have been equivocal across different global regions, studies in European youth have been fairly consistent in their findings with higher physical fitness in rural children and adolescents as compared with their peers [2,31,39,40,41]. In addition to natural processes of growth and maturation, prior exposure to and current PA levels are considered important determinants of physical fitness [42,43]. Associations between total PA and physical fitness, however, have only been low to moderate [44,45,46]. The limited association of PA and physical fitness could be attributed to the fact that PA is a multidimensional behavior with considerable daily variability, while physical fitness is a physiological or functional attribute [2]. Adolescents who are meeting current PA guidelines of at least 60 min of moderate-to-vigorous PA, nevertheless, have a three to eight times greater cardiorespiratory fitness than their sedentary peers, independent of the environment [47]. Various studies also showed higher PA levels in rural children as compared with their urban peers [40,48,49]. An improvement in physical fitness, however, requires prolonged engagement in a sufficient amount and intensity of PA. Accordingly, early engagement in PA is considered a critical component in the promotion of physical fitness [50].

Higher PA in rural children at younger ages has at least partially been attributed to a greater availability of safe outdoor spaces as there appears to be a direct association between time spent outdoors and PA in children [14,15,39]. In contrast, indoor time has been associated with more sedentary choices [15]. The selection of leisure pursuits can also be influenced by perceived safety, particularly in urban settings [2]. In fact, available research indicates a higher preference of indoor sports in urban children, whereas rural children were more comfortable playing outside [13]. More time spent outdoors, however, has been associated with greater improvements in physical fitness [51], which can be attributed to the higher intensity of activities performed outdoors as compared with indoor activities [52]. A comparison of physical education (PE) classes conducted indoors and outdoors also showed greater improvement in motor abilities with outdoor PE, although class time was similar for indoor and outdoor PE [53]. Higher PA levels during childhood, along with a greater amount of PA accumulated outdoors, can also explain higher physical fitness levels in rural adolescents as compared with their urban peers [2,54] despite their lower PA levels, particularly during the weekend [2,31,55]. A potential explanation for lower PA in rural adolescents could be a greater contribution of organized sports to total PA during adolescence, while free play becomes less important at older ages [56]. Although an urban environment potentially provides fewer open spaces for free play, which is an important contributor to PA during childhood, there are more opportunities for participation in organized sports, which potentially increases total PA in adolescents.

The results of this study further highlight the need for intervention strategies at young ages as differences in physical fitness became more pronounced with increasing age, already during the elementary school years. Particularly, normal weight children and girls in urban settings warrant special attention in the promotion of physical fitness, in addition to children and adolescents with overweight or obesity. Access to recreational facilities along with walkability and neighborhood safety have been shown to affect PA in youth [57]. Accordingly, home, school, streets, industrial areas, shops, park, and public spaces can contribute to or hinder PA. In addition to the built environment the perceived environment is of particular importance for groups at risk for low physical fitness, as outdoor activity appears to be a crucial component in the promotion of an active lifestyle [10,14,58]. A PA friendly environment provides external motivation for behavioral choices that include various activities to enhance physical fitness. Furthermore, living environment has been shown to affect biological aspects that are associated with physical fitness. Previous studies, for example, have shown that earlier maturation and increased body size in urban youth [3,4] also affect physical fitness [59].

Unfortunately, this study did not include additional assessments beyond anthropometric measurements that could determine biological maturation. There was also no information on total PA, sports participation, socioeconomic background, or perception of the environment. Given the voluntary participation of schools in the project, selection bias could also be present. In addition, participating children could have attended school in an urban area while living in a rural environment and vice versa. The cross-sectional nature of the study along with the narrow age range of the participants further limits the generalizability of the results beyond childhood and does not provide any information on potential tracking of physical fitness throughout childhood into adolescence. Furthermore, it should be considered that motivation during the fitness tests can differ among participants, which could affect the results. Given the large sample size, motivational differences, however, should have been mitigated. The large sample size should also have reduced a potential selection bias. Due to the homogeneity of the sample the influence of socioeconomic factors on the study outcomes should have been limited as well. In addition, the administration of multiple validated tests that assess various components of physical fitness by trained technicians should be considered a strength of this study.

## 5. Conclusions

Environmental conditions have been shown to affect various aspects in people’s lives including PA and physical fitness [60]. In particular, time spent outdoors is positively associated with PA and physical fitness and the built environment appears to be an important contributor to time spent outdoors [14,15]. Results of this study, along with data from other European countries, indicate that urban living environment increases the risk for low physical fitness in children [2,31]. Given the importance of physical fitness on general health and well-being, along with the association of physical fitness with an active lifestyle [20,24], intervention strategies targeting children living in urban areas are particularly important. Access to safe outdoor spaces that promote free play and exercise (e.g., public park, playground, and playing fields), facilitation of active transportation (e.g., sidewalks and bike lanes), and opportunities for organized sports for children at young ages are important aspects in environmental facilitation of PA that contributes to enhanced physical fitness. Additional research that considers specific living conditions (e.g., type of housing and number of occupants) and the factors that influence perception of the environment, however, is necessary to provide further information for the design of an activity permissive environment that promotes physical fitness in children, which would help in the promotion of an active lifestyle throughout adolescence and into adulthood.

## Figures and Tables

**Table 1:** Descriptive characteristics. Values are mean ± SD, except for overweight/obesity where prevalence is shown.

	Total Sample (N = 18,168)	Urban (N = 4118)	Rural (N = 14,050)	*p*-value
Overweight/obesity (%)	14.7%	15.8%	14.3%	0.015
Age (years)	8.4 ± 0.8	8.3 ± 0.7	8.4 ± 0.8	<0.001
Height (cm)	132.2 ± 7.0	132.0 ± 6.9	132.3 ± 7.1	0.027
Weight (kg)	29.8 ± 7.1	29.8 ± 7.3	29.8 ± 7.1	0.503
BMI percentile	51.8 ± 29.7	52.8 ± 29.9	51.5 ± 29.7	0.013
Vertical jump (cm)	19.9 ± 3.8	19.6 ± 3.8	20.0 ± 3.9	<0.001
Medicine ball push (cm)	351 ± 73	349 ± 72	351 ± 74	0.158
Tapping (# in 6 s)	45.0 ± 7.5	44.2 ± 7.5	45.2 ± 7.5	<0.001
10 m sprint (s)	2.28 ± 0.17	2.30 ± 0.17	2.27 ± 0.17	<0.001
Agility test (s)	20.0 ± 3.6	20.5 ± 3.8	19.8 ± 3.6	<0.001
Throw and catch (# in 30 s)	15.2 ± 7.7	14.5 ± 7.6	15.4 ± 7.7	<0.001
Stand-and-reach (cm)	1.7 ± 6.6	1.9 ± 6.8	1.6 ± 6.6	0.031
6 min run (m)	982 ± 134	961 ± 135	989 ± 133	<0.001

**Table 2:** Physical fitness by living area separately for girls and boys. Values are mean ± SD.

	GIRLS		BOYS	
	Urban (N = 2039)	Rural (N = 6803)	Urban (N = 2079)	Rural (N = 7247)
Vertical jump (cm) ^1,2^	19.0 ± 3.6	19.5 ± 3.7	20.1 ± 3.9	20.4 ± 3.9
Medicine ball push (cm)	321 ± 64	327 ± 66	372 ± 73	375 ± 75
Tapping (# in 6 s) ^1,2^	42.0 ± 7.2	43.3 ± 7.4	46.4 ± 7.1	47.0 ± 7.2
10 m sprint (s) ^1,2^	2.34 ± 0.17	2.30 ± 0.17	2.26 ± 0.16	2.24 ± 0.17
Agility test (s) ^1,2^	21.1 ± 3.7	20.3 ± 3.5	19.8 ± 3.8	19.4 ± 3.6
Throw and catch (# in 30 s) ^1^	11.8 ± 7.1	13.3 ± 7.4	17.1 ± 7.2	17.4 ± 7.4
Stand-and-reach (cm) ^1^	3.6 ± 6.6	3.2 ± 6.5	0.2 ± 6.5	0.1 ± 6.4
6 min run (m) ^1,2^	926 ± 122	956 ± 119	996 ± 138	1020 ± 138

^1^ significant urban–rural difference in girls, after adjusting for age and BMIPCT (*p* < 0.01); ^2^ significant urban–rural difference in boys, after adjusting for age and BMIPCT (*p* < 0.01).

**Table 3:** Physical fitness by living area separately for non-overweight and overweight/obese. Values are mean ± SD.

	Non-Overweight		Overweight/Obese	
	Urban (N = 3466)	Rural (N = 12040)	Urban (N = 652)	Rural (N = 2010)
Vertical jump (cm) ^1^	20.2 ± 3.5	20.5 ± 3.6	16.8 ± 3.2	17.0 ± 3.2
Medicine ball push (cm)	344 ± 60	346 ± 60	381 ± 80	381 ± 77
Tapping (# in 6 s) ^1^	44.6 ± 7.0	45.4 ± 7.0	43.2 ± 6.9	43.9 ± 6.9
10 m sprint (s) ^1,2^	2.27 ± 0.16	2.25 ± 0.15	2.42 ± 0.18	2.39 ± 0.19
Agility test (s) ^1,2^	19.9 ± 3.1	19.4 ± 3.1	23.3 ± 4.6	22.6 ± 4.5
Throw and catch (# in 30 s) ^1^	14.6 ± 6.4	15.3 ± 6.4	15.6 ± 6.5	16.0 ± 6.5
Stand-and-reach (cm)	1.9 ± 6.4	1.7 ± 6.4	1.3 ± 6.7	0.9 ± 6.7
6 min run (m) ^1,2^	985 ± 118	1008 ± 118	846 ± 124	873 ± 125

^1^ significant difference in non-overweight, after adjusting for age and sex (*p* < 0.01); ^2^ significnat difference in overweight/obese, after adjusting for age and sex (*p* < 0.01).
